# Left Heart and Systemic Arterial Circulation Air Embolus During CT-Guided Lung Biopsy

**DOI:** 10.7759/cureus.32402

**Published:** 2022-12-11

**Authors:** Christopher W Bailey, Kendal Angell, Ayub Khan, Jeffrey Elbich

**Affiliations:** 1 Interventional Radiology, Virginia Commonwealth University School of Medicine, Richmond, USA; 2 Medicine, West Virginia School of Osteopathic Medicine, Lewisburg, USA

**Keywords:** hemodynamic management, transthoracic needle biopsy, interventional radiology, lung cancer, lung biopsy, air embolism

## Abstract

A transthoracic needle biopsy (TTNB) of the lung, commonly referred to as a “lung biopsy,” is a commonly performed procedure in Interventional Radiology. It is usually associated with well-known risks including pneumothorax and hemothorax. One of the rare and lesser-known risks of TTNB, however, is a phenomenon called an air embolism. The term “air embolism” alone may be somewhat ambiguous, as it could indicate i) air entering the systemic veins, or ii) air entering the pulmonary veins. Here, we present a case of an air embolus entering the pulmonary veins. The pulmonary veins naturally drain into the left side of the heart (left atrium and ventricle) which provides oxygenated blood to the major arteries of the body including the coronary, carotid, and major abdominal visceral branches. Therefore, an air embolism in this vasculature can lead to potentially devastating hemodynamic consequences downstream.

## Introduction

Lung cancer is the leading cause of cancer-related deaths worldwide in both men and women [1]. Oftentimes lung nodules or masses are found incidentally on radiologic imaging studies indicated for a separate purpose. In many instances, these findings have features indicative of malignancy. The treatment for these types of lung nodules typically requires a tissue diagnosis, necessitating a transthoracic needle biopsy (TTNB) of the lung. TTNB is a common procedure performed by Interventional Radiologists, characterized by being minimally invasive compared to open surgical methods. By obtaining biopsy samples through TTNB, it is possible to identify specific characteristics of the malignant tissue and then determine a treatment strategy tailored to that unique malignancy [2].

TTNB procedures are generally safe procedures with major complications consistent with guidelines, including pneumothorax requiring intervention, hemothorax, and death, demonstrating risks as low as 5.7% [2]. While many practitioners know about these possible complications, an awareness of the rare, but potentially devastating complication of an air embolus, must not be overlooked. These emboli have the potential to enter the left heart (systemic arterial circuit) as described in our case, with the possibility of causing further downstream cardiopulmonary instability and ischemia [3,4]. In the authors’ opinion, an air embolism involving the left heart is an unfortunate and unforeseeable complication that can accompany any TTNB procedure.

## Case presentation

A 72-year-old woman with a history of osteopenia presented to her neurosurgeon for chronic back pain. She went on to receive a computed tomography (CT) scan of her Thoracic spine at the physician’s request. The images did not reveal any significant spinal pathology; however, an 8 mm x 7 mm nodule with irregular margins in the left lower lobe of her lung presented incidentally. Several smaller nodules were also partially visible and incompletely evaluated, prompting an additional dedicated chest CT with IV contrast. The dedicated chest CT images confirmed a 9-mm spiculated pulmonary nodule in the left lower lobe amongst several smaller nodules, which was overall concerning for malignancy.

**Figure 1:**
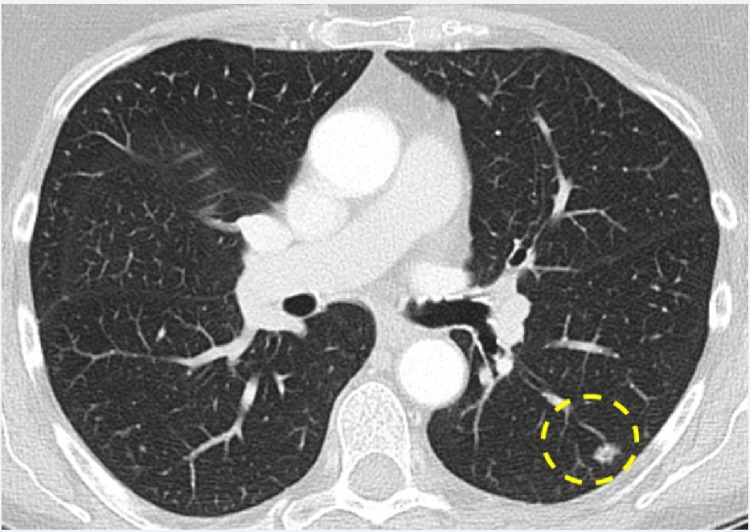
Enhanced CT of the chest with lung algorithm Axial image through the level of the left lower lobe nodule (perforated yellow circle). Note: patient in supine position.

Subsequent consultation with cardiothoracic surgery generated a decision to repeat CT Chest imaging in three months. The follow-up scan redemonstrated the spiculated nodule (Figure 1) but with an increased volume (now 9.4 mm long axis, by 6.7 mm short axis). Referral to Interventional Radiology (IR) for TTNB of the spiculated left lower lobe pulmonary nodule was then requested.

**Figure 2:**
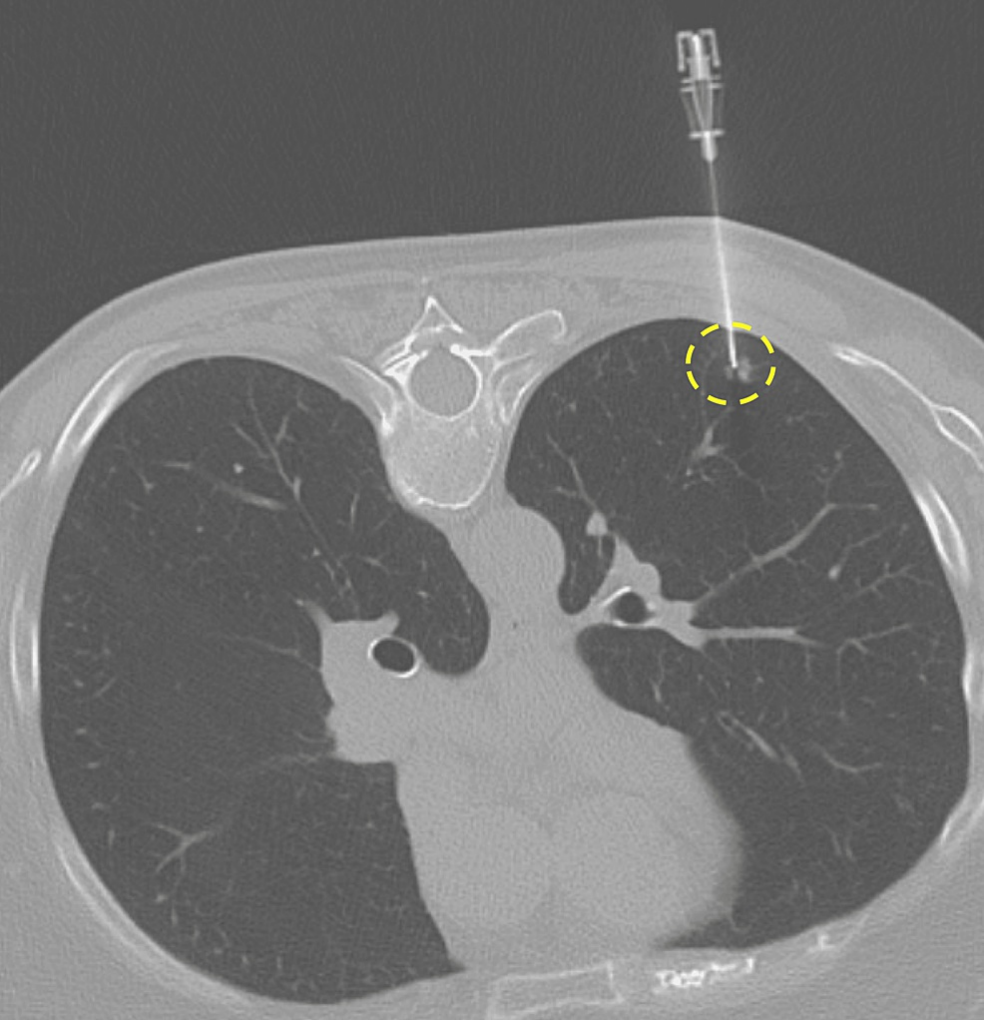
Unenhanced CT of the chest with lung biopsy algorithm The axial image through the level of the left lower lobe nodule (perforated yellow circle) with a biopsy trocar needle in place. Note: patient in prone position.

To target the pulmonary nodule for biopsy, CT guidance was utilized with the patient placed in a prone position. During the procedure, cardiopulmonary monitoring of the patient was achieved via blood pressure, heart rate, pulse oxygenation, and end-tidal capnography. In addition, intravenous conscious sedation with Fentanyl and Midazolam was used. To begin the procedure, a 19-gauge coaxial trocar needle was advanced percutaneously toward the suspicious spiculated lung nodule (Figure 2). The position was confirmed with intermittent axial CT images. Once the target was reached, a compatible adjustable Coaxial TEMNO 20-gauge biopsy device (Merit Medical, Jordan UT) was passed through the trocar needle, and a biopsy of the spiculated nodule was performed. A total of four 20-gauge core needle biopsy samples were obtained. Pathology personnel was present for the biopsy and after the “touch prep” formation of microscopic slides, they deemed the samples adequate. Afterward, the biopsy needle system was removed without complications, and a bandage was placed over the site. Up to this point, the patient tolerated the sedation and lung biopsy procedure without issues.

**Figure 3:**
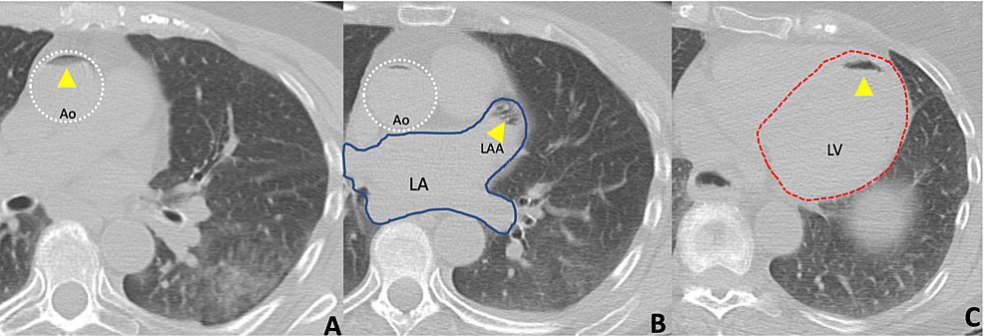
Unenhanced CT of the chest with lung biopsy algorithm The axial image through the ascending aorta (perforated white circle) shows an air-fluid level (yellow arrowhead) (A).  The axial image through the left atrium (solid blue outline) shows an air-fluid level (yellow arrowhead) in the left atrial appendage (LAA) (B). The axial image through the left ventricle (perforated red outline) shows an air-fluid level (yellow arrowhead) in the anterior most portion of the left ventricle (C). Note: patient in supine position.

**Figure 4:**
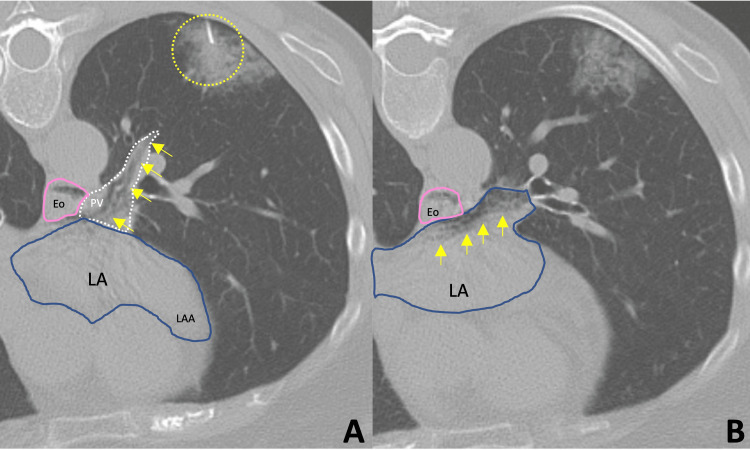
Unenhanced CT of the chest with lung biopsy algorithm The axial image through the left pulmonary vein (perforated white outline) shows air mixing with fluid/blood (yellow arrowheads) (A). Also, there is an air-fluid level in the esophagus (pink outline) which is expected. A lung biopsy needle at the biopsy site is also depicted (yellow perforated circle). The axial image through the left atrium shows air mixing with fluid/blood (yellow arrowheads) (B). Note: patient in prone position.

Once supine, the patient complained of feeling faint, endorsing left arm weakness and paresthesia. She quickly became hypoxic and bradycardic, prompting a rapid chest CT. The scan demonstrated air densities within the left ventricle and ascending aorta, including the right innominate artery (Figures 3A-3C). A review of imaging during the procedure showed air within the left pulmonary veins, left atrium, and a presumed bronchiole-pulmonary vein fistula (Figures 4A, 4B). A code blue was initiated, and the patient was positioned with her head angled downward, laying in right lateral decubitus. A non-rebreather mask was placed and an EKG was performed, demonstrating new-onset atrial fibrillation and NSTEMI. A stroke alert then ensued, resulting in a non-contrast head CT, a CTA of the head and neck with perfusion, and a transthoracic echocardiogram. Afterward, she was transferred to the pulmonary intensive care unit for further management, maintaining left lateral decubitus and Trendelenburg when possible.

Her weakness and paresthesia from the inciting event resolved within minutes. While in intensive care, however, she went on to develop hemoptysis, chest pain, nausea, and vomiting. Brain imaging from the stroke alert was unremarkable, but her echocardiogram demonstrated decreased left ventricular ejection fraction (LVEF), and anterior wall motion abnormalities (WMA). She also had an elevated troponin believed to be from the passage of air embolism through the coronary arteries in the left anterior descending artery (LAD) distribution. She remained in intensive care overnight.

Fortunately, the following day, she experienced a significant clinical improvement, including cessation of her nausea, hemoptysis, and chest pain, with a spontaneous resolution of her atrial fibrillation to normal sinus rhythm. She was then transferred to a step-down medical unit on an aspirin, statin, and metoprolol regimen. The next day she was discharged with a follow-up echo and brain MRI, each of which was unremarkable, exhibiting normalization of the LVEF and WMA.

## Discussion

While generally rare, an air embolism event is a potentially lethal consequence of several procedures across many medical specialties, including TTNB [3]. Most clinicians and proceduralists are aware of air embolus related to central venous access procedures, in which a connection between atmospheric air and the central veins occurs through a needle or central line placement. In this case, atmospheric air enters the venous circulatory system through a conduit (access needle or unclamped central line) when a large negative intrathoracic force (or vacuum) is created by the diaphragmatic movement during inspiration. In other words, when the central venous pressure (CVP) is lower than atmospheric pressure (P_ATM_), a simple deviant connection between a needle and a vein can produce a pathway for the higher P_ATM_ to travel into the patient’s lower-pressure vasculature [4].

In our case, atmospheric air is entering the arterial circuit. The air within the arteries acts as a space-occupying mass, akin to a blood clot, which can manifest as a stroke, myocardial infarction, mesenteric ischemia, or cold limb. In particular, the incidence of air embolism during TTNB is limited, but reported data has shown a radiological incidence of 0.21%-3.80% and a mortality incidence of 0.16% [5,6].

Left heart and systemic arterial circulation air embolus during CT-guided lung biopsy are not intuitive. It is hypothesized to be introduced via a fistulous communication between a pulmonary vein and an adjacent bronchiole. The fistulous connection is thought to be iatrogenic and forms traumatically during the advancement of the biopsy needle through the lung parenchyma/ target lesion. The needle creates a bronchiole-to-pulmonary venous fistula, where the intrabronchial air can theoretically enter pulmonary venous circulation. Coughing, straining, or Valsalva maneuvers during the needle advancement increase this risk of fistula, by causing an increase in pressure within the lung [7-9].

When air emboli occur during TTNB, signs of cardiopulmonary or neurological decompensation (dyspnea, chest pain, bradycardia, hypotension, ventricular fibrillation, or changes in GCS) can indicate cardiac or cerebral ischemia. If symptoms are quickly recognized and appropriate intervention administered, these events can be non-fatal and without long-term morbidity [5,10].

The ultimate prognosis of an air embolism often depends on the volume of air that enters circulation and its rate of entry. Air entering the left side of the heart (as in our patient) cannot be dissipated in the lungs, causing air to accumulate in the left atrium or ventricle and impede filling. The air embolus may also travel from the left ventricle to the aorta, occluding any of the downstream peripheral vessels [11-13].

In any case of air embolism regardless of the mechanism, supportive treatment and closure of any conduit between the atmosphere and the vascular system should be implemented immediately. At a minimum, the patient should be given 100% oxygen. Some data suggest placing the patient in the right lateral decubitus position; whereas others recommend the Trendelenburg position. The goal of the positions is to prevent the air from leaving the left ventricle and traveling into systemic circulation through the aorta [5]. Endovascular intervention, including catheter-assisted aspiration of the air embolus can be controversial, but may be attempted in some cases. Hyperbaric oxygen therapy has sometimes proven helpful in treatment, decreasing the air bubble size by increasing the P_ATM_. Ideally, this hyperbaric therapy should be initiated within four to six hours of symptom presentation, so factors such as feasibility, availability, and individual patient needs should be evaluated when considering this option [12-14]. 

## Conclusions

A lung biopsy is a commonly requested procedure that carries well-known associated risks such as pneumothorax or hemothorax. An air embolism, however, remains a lesser-known rare complication of the procedure, that carries potentially catastrophic consequences. In particular, it may occur in the left side of the heart, with the potential to travel through the aorta causing severe downstream hemodynamic collapse. While the complication is rare, it is important that close cardiopulmonary monitoring of the patient is maintained both during and after any TTNB procedure. Prompt recognition of a left-sided air embolus is crucial for expedient treatment, which can significantly reduce its associated morbidity and mortality. It is imperative that providers on all levels of care be aware of this risk and its potential consequences when their patients undergo seemingly routine lung biopsies.
